# Macrophage polarization toward M1 phenotype through NF-κB signaling in patients with Behçet’s disease

**DOI:** 10.1186/s13075-022-02938-z

**Published:** 2022-11-04

**Authors:** Xiuhua Wu, Zhimian Wang, Jing Shi, Xin Yu, Chaoran Li, Jinjing Liu, Fengchun Zhang, Hua Chen, Wenjie Zheng

**Affiliations:** 1 Department of Rheumatology and Clinical Immunology, Chinese Academy of Medical Sciences & Peking Union Medical College; National Clinical Research Center for Dermatologic and Immunologic Diseases (NCRC-DID), Ministry of Science & Technology; State Key Laboratory of Complex Severe and Rare Diseases, Peking Union Medical College Hospital (PUMCH); Key Laboratory of Rheumatology and Clinical Immunology, Ministry of Education, Beijing, 100730 China; 2 Department of Rheumatology and Immunology, Tianjin Medical University General Hospital, Tianjin, 300052 China; 3 Department of Rheumatology, Zhongshan Hospital, Fudan University, Shanghai, 200032 China

**Keywords:** Behçet’s disease, Macrophage polarization, Phagocytosis, Th1 differentiation, NF-κB pathway

## Abstract

**Background:**

Macrophages are key innate immune cells implicated in the pathogenesis of Behçet’s disease (BD), and macrophage polarization plays a pivotal role in inflammatory response. This study aimed to investigate the role of BD serum on the phenotypes and functions of macrophage polarization.

**Methods:**

BD or HC serum-treated human monocyte-derived macrophages (HMDMs) were examined M1/M2 phenotypes using flow cytometry and ELISA. The phagocytic capacity of HMDMs and CD4^+^T cell differentiation facilitated by HMDMs were measured by flow cytometry. Transcriptome analysis of BD and HC serum-stimulated HMDMs was conducted to identify differentially expressed genes. NF-κB signaling was examined using western blot to explore the mechanism of macrophage polarization induced by BD serum.

**Results:**

BD serum-treated macrophages expressed a higher level of CD86, IL-12, and TNF-α and a lower level of CD163, which were compatible with the M1-like phenotype. Furthermore, BD serum-treated macrophages showed enhanced phagocytic capacity and promoted more Th1 cell differentiation. Sixty-one differentially expressed genes were identified between BD and HC serum-treated macrophages and were enriched in NF-κB signaling. BD serum-treated macrophages showed upregulated p-p65 and downregulated IκBα, and NF-κB inhibitor attenuated BD serum-stimulated M1-like phenotype.

**Conclusions:**

BD serum promoted macrophage polarization toward a proinflammatory M1-like phenotype through NF-κB signaling and potentially facilitated inflammation in BD. M1 polarized macrophages may be a potential therapeutic target for BD.

**Supplementary Information:**

The online version contains supplementary material available at 10.1186/s13075-022-02938-z.

## Background

Behçet’s disease (BD) is a chronic inflammatory systemic vasculitis, characterized by recurrent oral /genital ulcers, skin lesions, and organ involvements, including uveitis, cardiovascular, gastrointestinal, and central nervous systems [1]. BD is prevalent (20~602 per 100,000) in Silk road countries spanning from China to the Mediterranean area [2]. BD is typically onset at young and middle aged [3] and progressively and recurrently impairs physical, mental, and social capacities, Quality of Life (QoL), and even life span, causing considerable financial costs to society and individuals. The pathogenesis of BD remains elusive, where genetic predisposition and environmental stressors might play together, leading to overactivation of the immune system and inflammatory damage of multi-systems [4], mainly manifested by enhanced inflammatory responses and overexpression of proinflammatory cytokines [5]. Current therapies include glucocorticoids, immunosuppressive agents, and emerging TNF inhibitors. Therefore, an in-depth understanding of the pathogenesis and the development of new therapeutic targets are essential for improving the prognosis and reducing the disease burden of BD.

Macrophages are key innate immune cells that initiate adaptive immunity by antigen presentation and cytokine production. Macrophages play a critical role in inflammation through phagocytosis and the production of proinflammatory cytokines and chemokines. Macrophages are categorized into two subtypes: classically activated macrophages (M1 macrophages) and alternatively activated macrophages (M2 macrophages). M1 macrophages are induced by lipopolysaccharides (LPS) and interferon-γ (IFNγ) and produce proinflammatory cytokines, while M2 macrophages are induced by IL4, IL-10, or IL-13 and exert anti-inflammatory and promote injury healing and tissue repair [6]. Macrophage polarization, defined as phenotypic and functional plasticity of macrophages, is potentially implicated in autoimmune diseases, such as systemic lupus erythematosus (SLE), rheumatoid arthritis (RA), Sjögren’s syndrome (SS), and inflammatory bowel diseases (IBD) [7, 8].

Recent studies suggest M1 macrophage polarization in BD [9]. TNF-α, IL-1β, IL-6, IL-8, and IL-12 are major proinflammatory cytokines secreted by M1 macrophages and are elevated in BD [10]. M1 macrophages are observed in herpes simplex virus (HSV)-induced BD mouse model [11], and BD serum induces healthy donor monocytes to polarize to M1 macrophage in vitro [12]. However, the potential function and mechanism of M1 macrophage polarization in BD remains largely unknown. GWAS study identifies CCR1 and IL10 as risk loci of BD, which might promote M1 macrophage [13]. In this study, we performed phenotypic and functional investigations on BD serum-induced M1 macrophage polarization. Furthermore, we performed transcriptome analysis on macrophages to explore potential mechanisms driving M1 activation of macrophages.

## Methods

### Patients and controls

Forty-five treatment-naïve active BD patients (27 males, age 33.0 ± 11.2 years, disease duration 94.4 ± 78.4 months) were recruited from Peking Union Medical College Hospital (PUMCH) between March 2014 and December 2019 (Supplemental Table S1). All BD patients fulfilled the International Criteria for Behçet’s Disease (ICBD) [14], and active BD was defined as Behçet’s Disease Current Activity Form (BDCAF) score≥1 or with elevated erythrocyte sedimentation rate (ESR)/high-sensitivity C-reactive protein (hsCRP). Forty-five gender-and age-matched healthy volunteers (25 males, mean age 36.8 years) were enrolled as healthy controls (HC). Serum samples of BD patients and paired HC were collected and stored at −80°C until use within 2 years. Twenty-nine paired BD and HC samples were used for the phenotype (*n*=12) and functional (*n*=17) analysis, 4 paired samples for bulk RNA-seq analysis, and the rest 12 paired for verifying the activated signaling pathway (Supplemental Table 1.1 and 1.2). The study was approved by the institutional review board of PUMCH, and written informed consent was obtained from all subjects in accordance with the Declaration of Helsinki.

### Cells

Monocytes were isolated from HC peripheral blood mononuclear cells (PBMCs) with CD14^+^ MicroBeads (Miltenyi Biotec) with a purity >95% by flow cytometry (Supplemental Figure S1A). Monocytes (1.5×10^6^ cells/mL) were seeded onto 24- or 48-well plates and were incubated in a complete DMEM medium supplemented with M-CSF (50ng/ml, Sigma) for 7 days to differentiate into adherent HMDMs. Complete DMEM contains DMEM (Gibco), 10% fetal bovine serum (FBS, Gibco), and penicillin and streptomycin (Gibco). The purity of HMDMs was > 80% measured by intracellular CD68 on day 7 (Supplemental Figure S1B). Naive CD4^+^ T cells were isolated from HC PBMCs using naive CD4^+^ T cell isolation kit II (Miltenyi Biotec) with a purity > 90% (Supplemental Figure S1C).

### Macrophage polarization

Resting (M0) macrophages were defined as HMDMs without additional stimulation. M0 were stimulated with LPS (100ng/ml) plus IFNγ (20ng/ml), or IL-4 (20ng/ml) plus IL13 (20ng/ml) for 48 h to differentiate into M1 or M2 macrophages, respectively. M0 were also treated with 10% BD or HC serum for 48 h. HMDMs were harvested using 0.25% trypsin-EDTA digestion for 10 min at 37°C. Surface CD86, CD163, and CD206 were measured with flow cytometry, and supernatant TNF-α, IL-12, CXCL2 and CXCL3 were measured by enzyme-linked immunosorbent assay (ELISA) (BioLegend or MultiSciences).

### Phagocytosis assay

M0, M1, M2, and BD or HC serum-treated HMDMs were incubated in PBS with 1% BSA at 4°C for 30 min. After washing in PBS, cells were incubated with FITC-Dextran (Santa Cruz) and shaken at room temperature for 30 min. Cells were harvested and washed twice with PBS, 1% BSA. Intracellular FITC-dextran was determined by flow cytometry.

### Macrophage-dependent T cell differentiation

M0, M1, and M2 conditions or BD and HC serum-pretreated HMDMs (5×10^4^) were harvested and incubated with 2.5×10^5^ naïve CD4^+^T cells in 48-well plates, and polarized in 500μl complete DMEM medium with plate-bound anti-CD3 (5 μg/ml, BD Biosciences), soluble anti-CD28 (5 μg/ml, BD Biosciences), anti-IL4 (5 μg/ml, BioLegend), and IL-2 (10 ng/ml, BioLegend) for 5 days. Before harvest, T cells were stimulated with Leukocyte Activation Cocktail (BD Bioscience) for 4 h, and IFNγ^+^ and T-bet^+^ CD4^+^ T cells were measured by flow cytometry.

### Flow cytometry

For macrophages staining, macrophages were pretreated with Fc Receptor Blocking Solution (1:20, BioLegend) for 10 min at room temperature and were stained with surface antibodies and Ghost Dye (1:1000, Tonbo Biosciences) at 4°C for 30 min in dark. Macrophages were also fixed and permeabilized with Fixation/Permeabilization Solution (BD Biosciences) and were stained with anti-CD68 (Y1/82A, BioLegend). For T cell intracellular staining, cells were fixed and permeabilized with Foxp3/Transcription Factor Staining Buffer (eBioscience), and intracellular cytokine/ nuclear transcription factor staining was performed according to the manufacturer’s protocol.

The following monoclonal antibodies (mAbs) were used: FITC anti-CD86 (BU63, BioLegend), PE anti-CD163 (GHI/61, BioLegend), APC anti-CD206 (15-2, BioLegend), and PerCP-Cy55 anti-CD68 (Y1/82A, BioLegend), FITC anti-CD4 (A161A1, BioLegend), PE-Cy7 anti-IFNγ (B27, BioLegend), PerCP-Cy55 anti-IL-17A (BL168, BioLegend), Alexa 647 anti-T-bet (O4-46, BD Biosciences), and PE anti-RORγt (AFKJS-9, BD Biosciences). Appropriately matched isotype control mAb to each antigen-specific mAb was used for control.

The stained cells were immediately analyzed on FACSAria II (BD Biosciences) flow cytometer, and data analysis was performed with the FlowJo software (Tree Star).

### Bulk RNA-seq data analysis

Total RNA was extracted from BD or HC serum-treated HMDMs using TRIzol (Invitrogen). Sequencing libraries were generated with NEBNext® UltraTM RNA Library Prep Kit for Illumina® (NEB, USA) and qualified by the Agilent Bioanalyzer 2100 system. The clustering of the index-coded samples was performed using TruSeq PE Cluster Kit v3-cBot-HS (Illumia). The library preparations were sequenced on an Illumina Hiseq platform, and 125 bp paired-end reads were generated.

Raw data in FASTQ format were first processed through in-house Perl scripts. In addition, the Q20, Q30, and GC contents of the clean data were calculated. All downstream analyses were based on clean, high-quality data. A reference genome index was built, and paired-end clean reads were aligned to the reference genome using HISAT2 (v2.0.5). For quantification of gene expression levels, featureCounts (v1.5.0) was applied to count the number of reads mapped to each gene. The FPKM value of each gene was then calculated based on the length of the gene and the number of reads mapped to that gene.

The count matrix was input into DESeq2 (v1.30.0) [15] and fitted for a general linear model with a negative binomial distribution. To calculate DEGs, batch effect was corrected within DESeq2 and DEGs were identified by the functions DESeq with the adjusted *p* <0.05 (Wald test and Bonferroni correction). For PCA and heatmap demonstration, the matrix was corrected by R package sva to remove batch effect and normalized by Function rlog in DESeq2. PCA was performed for top 2000 variable genes based on variance, and the results were visualized with the function pca in R package PCAtools.

### Pathway enrichment analysis

The enriched pathways were assessed by hypergeometric testing in the Gene Ontology (GO) and Kyoto Encyclopedia of Genes and Genomes (KEGG) databases based on DEGs by R package clusterProfiler (v3.0.4) [16]. Significantly enriched pathways were determined with a cutoff of a Benjamini–Hochberg-corrected *p* < 0.05.

### Gene signature analysis

Gene signatures were downloaded from the KEGG database and C7 gene sets of the MSigDB Collections [17], including *GSE16385_UNTREATED_VS_12H_IL4_TREATED_MACROPHAGE_DN*, *GSE9509_LPS_VS_LPS_AND_IL10_STIM_IL10_KO_MACROPHAGE_30MIN_DN*, and *GSE25088_CTRL_VS_IL4_STIM_STAT6_KO_MACROPHAGE_UP* gene sets from the previous studies [18, 19] which were relevant to our study. Significantly enriched pathways were determined with the cutoff of *p* value <0.05, Benjamini–Hochberg-corrected *p* < 0.25, and absolute value of negative normalized enrichment score (NES) >1.

### Western blot

HMDMs were stimulated with BD serum or HC serum for 0, 15, 30, and 60 min. Total proteins of 1–2×10^6^ HMDMs were extracted with Minute Total Protein Extraction Kit (Invent Biotechnologies) and were quantified by BCA Assay Kit (Pierce). Proteins were loaded and electrophoresed on a 4–20% SDS-PAGE gel and were subsequently transferred to a PVDF membrane (Millipore). The membrane was blocked with tris-buffered saline-Tween 20 (TBST) containing 5% non-fat milk for 1 h at room temperature followed by incubation overnight with anti-NF-κB p65 rabbit antibody, anti-Phospho-NF-κB p65 rabbit antibody, anti-IκBα rabbit antibody, anti-JAK1 mouse antibody, anti-Phospho-JAK1 rabbit antibody, anti-STAT1 rabbit antibody, anti-Phospho-STAT1 rabbit antibody or anti-β-actin rabbit antibody (Cell Signaling Technology) at 4°C. The membrane was washed three times and incubated with HRP-conjugated secondary antibodies (EASYBIO) for 1 h at room temperature. The proteins were visualized using a ChampChemi Multiplex Fluorescence /Chemiluminescence Imager (Sage Creation Science), and the optical density data were analyzed using ImageJ software. β-actin was used as the endogenous control.

### Statistical analysis

Quantitative data were expressed as mean ± standard deviation (SD) or median (range). Categorical variables were represented as frequencies and percentages. Student’s *t* test was used for comparing two groups. Multiple group comparisons were analyzed using one-way ANOVA and two-way ANOVA (normally distributed data) or Kruskal-Wallis test (non-normally distributed data). A two-sided *p* value < 0.05 was considered statistically significant. Analyses were performed with SPSS V.26 (SPSS, USA).

## Results

### BD serum promotes M1-like macrophage polarization

**Fig. 1:**
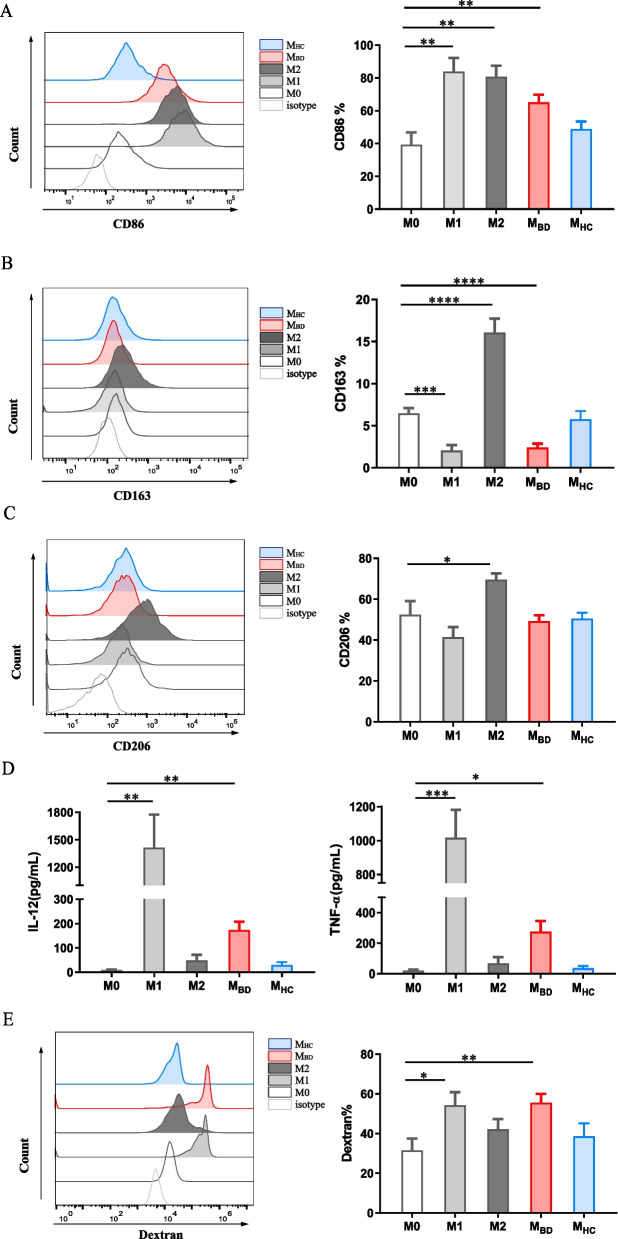
BD serum promotes M1-like macrophage polarization. Resting macrophages (M0) were stimulated with M1 condition (100ng/ml LPS+ 20ng/ml IFNγ), M2 condition (20ng/ml IL-4+ 20ng/ml IL-13), BD serum or HC serum for 48 h. **A–C** Representative histograms (left) and summary (right) of CD86, CD163 and CD206 expression level of macrophages stimulated with M0 (*n*=6), M1 (*n*=6), and M2 (*n*=6) conditions, as well as BD (*n*=12) serum and HC (*n*=12) serum. Data were expressed as mean±SD and were analyzed using one-way ANOVA. **D** IL-12 and TNF-α production by macrophages stimulated with M0 (*n*=6), M1 (*n*=6), and M2 (*n*=6) conditions, as well as BD (*n*=12) serum and HC (*n*=12) serum. Data were expressed as mean±SD and were analyzed using Kruskal-Wallis test. **E** Representative histograms (left) and summary (right) of dextran uptake by macrophages stimulated with M0 (*n*=7), M1 (*n*=7), M2 (*n*=7) conditions, and BD (*n*=9) serum and HC (*n*=9) serum. Data were expressed as mean±SD and were analyzed using one-way ANOVA. *, *p*<0.05; **, *p*<0.01; ***, *p*<0.001, ****, *p*<0.001. M_BD_, BD serum-treated macrophages; M_HC_, HC serum-treated macrophages

To explore the phenotype of BD serum-stimulated macrophages, we treated HMDMs with BD or HC serum as well as M0, M1, or M2 conditions. The phenotypes of macrophages are regulated by complex, dynamic environments, making it unlikely to define myeloid cell heterogeneity with a limited number of markers. Therefore, M1 and M2 macrophages were defined using CD86, CD163, CD206, IL-12, and TNF-α as previously described [20–22], to distinguish M1 and M2 macrophages more accurately. We verified a higher level of CD86 (M0: 39.3 ± 18.3%, M1: 84.0 ± 20.2%, M2: 80.0 ± 16.4%) on M1 and M2 macrophages, higher levels of CD163 (16.1±3.4% vs 6.5±2.0%, *p*<0.0001) and CD206 (69.6 ± 7.4% vs 52.5 ± 16.1%, *p*<0.05) on M2 macrophages, a lower level of CD163 (2.0 ± 1.8% vs 6.5 ± 2.0%, *p*<0.05) on M1 macrophages, and higher levels of IL-12 (1612.0 ± 876.4 vs 8.3 ± 4.3 pg/ml, *p*<0.001) and TNF-α (994.8 ± 334.0 vs 22.3 ± 14.0 pg/ml, *p*<0.001) produced by M1 macrophages. Consistent with M1 macrophages, BD serum-treated macrophages expressed a higher level of CD86 (65.1 ± 16.1% vs 39.3 ± 18.3%, *p*<0.05), a lower level of CD163 (2.4 ± 1.6% vs 6.7 ± 2.1%, *p*<0.01), and produced higher levels of IL-12 (164.0 ± 100.0 vs 8.3 ± 4.3 pg/ml, *p*<0.01) and TNF-α (253.1 ± 205.2 vs 22.3 ± 14.0 pg/ml, *p*<0.05) than M0 macrophages, which were not observed in HC serum-treated macrophages (Fig. 1A–D, Supplemental Figure S2 and S3).

Furthermore, we confirmed the enhanced cellular dextran uptake in M1 macrophages (M0: 31.5 ± 15.7%, M1: 54.3 ± 17.2%, M2: 42.2 ± 13.5%) as well as in BD serum-treated macrophages (55.6 ± 13.3% vs 31.5 ± 15.7%, *p*<0.05) but not HC serum-treated macrophages (Fig. 1E, Supplemental Figure S2D). Together, these findings suggest BD serum-induced macrophage polarization toward M1-like phenotype.

### BD serum stimulated macrophages to facilitate Th1 cell differentiation

**Fig. 2:**
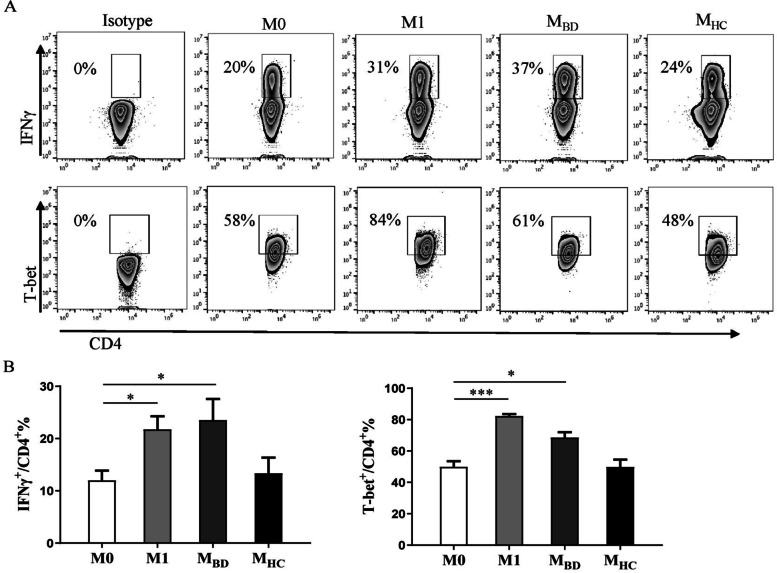
BD serum-treated macrophages facilitate Th1 differentiation. Naive CD4+ T cells were incubated with M0, M1, M2, BD serum- and HC serum-treated macrophages in Th1 condition (5μg/ml anti-CD3, 5μg/ml anti-CD28, 5μg/ml anti-IL-4, and 10ng/ml IL-2) for 5 days. **A** Representative flow cytometry plots and **B** summary of IFNγ and T-bet [*n*(M0)=3, *n*(M1)=3, *n*(M2)=3, *n*(M_BD_)=5, *n*(M_HC_)=5] expression levels in CD4+ T cells. Data were shown as mean±SD. *, *p*<0.05; **, *p*<0.01; ***, *p*<0.001, ****, *p*<0.001 by one-way ANOVA. M_BD_, BD serum- treated macrophages; M_HC_, HC serum- treated macrophages

Since macrophages are antigen-presenting cells promoting CD4^+^ T helper (Th) cell differentiation [23], we then investigated the potential differentiation of effector T cells assisted by BD serum-treated macrophages. We incubated naive CD4^+^ T cells with M0, M1, M2, BD, or HC serum-treated macrophages and observed that M1 macrophages and BD serum-treated macrophages (M0: 51.6 ± 7.1%, M1: 82.1 ± 8.8%, M_BD_: 66.7 ± 5.3%) promoted more IFNγ^+^CD4^+^ T cell differentiation than M0 macrophages (Fig. 2, Supplemental Figure S4). Meanwhile, M1 macrophages and BD serum-treated macrophages (M0: 40.6 ± 8.2%, M1: 73.6 ± 6.8%, M_BD_: 60.8 ± 9.5%) promoted T-bet expression (Fig. 2, Supplemental Figure S4 and S5). Additionally, BD serum-treated macrophages also promoted Th17 differentiation (Supplemental Figures S6). Collectively, these data suggested that BD serum-treated macrophages and M1 macrophages promoted Th1 differentiation.

### Transcriptome analysis of BD and HC serum-treated macrophages

**Fig. 3:**
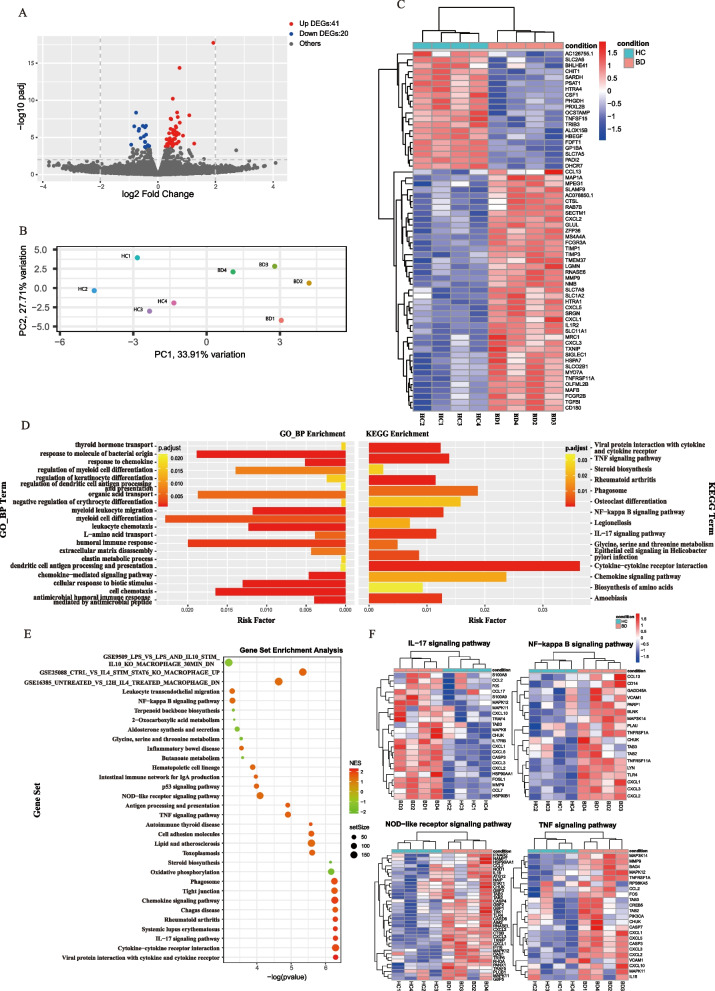
Transcriptome analysis of BD serum- and HC serum-treated macrophages. HMDMs were stimulated with serum from four treatment-naïve active BD patients and matched healthy volunteers for 48 h, and total RNA was extracted for RNA-seq analysis. **A** Principal component analysis (PCA) of BD serum-treated and HC serum-treated macrophages. **B** Volcano plot of upregulated (red, *n*=41) and downregulated (blue, *n*=20) DEGs in BD serum-treated macrophages compared with HC serum-treated macrophages. **C** Heatmap of DEGs between BD serum- and HC serum-treated macrophages. **D** GO biological process enrichment analysis and KEGG enrichment analysis between BD serum- and HC serum-treated macrophage. **E, F** Dot plots (left) showed Gene Set Enrichment Analysis (GSEA) of BD serum- and HC serum-treated macrophage. Representative enriched gene sets were illustrated by heatmap (right). DEGs, differentially expressed genes; GO, gene ontology; KEGG, Kyoto Encyclopedia of Genes and Genomes

To gain insights into the mechanism of BD serum-induced M1-like macrophage polarization, we next performed transcriptome analysis on macrophages stimulated with BD and HC serum (*n*=4). Principal component analysis (PCA) of the top 2000 variable genes showed distinct transcriptional patterns between two conditions (Fig. 3A). We identified 41 upregulated and 20 downregulated differentially expressed genes (DEGs, Fig. 3B,C and Supplemental Table S2), which showed a transcriptionally active gene signature in BD serum-induced macrophages. GO biological process and KEGG analysis revealed enriched migration and chemotaxis-related gene sets (Fig. 3D), including upregulated *CXCL1*, *CXCL2*, *CXCL3*, *CXCL5*, and *CCL13* (Fig. 3C). We validated that BD serum-treated macrophages produced more CXCL2 and CXCL3 (Supplemental Figure S7), which are characteristic of classical M1 macrophage activation [24]. KEGG and GSEA analysis showed enhanced inflammatory immune responses in BD serum-induced macrophages, including phagosome, IL-17 signaling pathway, and TNF-signaling pathway (Fig. 3D–F), which was confirmed by the phagocytosis test (Fig. 1E), enhanced Th17 differentiation (Supplemental Figure S6), and overproduction of TNF-α in BD serum-treated macrophages (Fig. 1D), respectively. A response to a molecule of bacterial origin (Fig. 3D) was implicated in BD serum-induced macrophages, supporting infections as a trigger of BD [25]. The enriched Nod-like receptor pathway and NF-κB pathway (Fig. 3D–F) suggested enhanced innate immune responses in BD serum-induced macrophages. Finally, we compared our gene signature with previous studies [18, 19] and found it demonstrated an opposite pattern from M2 polarization conditions such as IL-4 and IL-10 stimulation (Fig. 3E). Taken together, the transcriptome analysis suggested that BD serum promoted macrophage polarization toward a proinflammatory M1-like phenotype.

### BD serum polarize macrophages through the NF-κB pathway

**Fig. 4:**
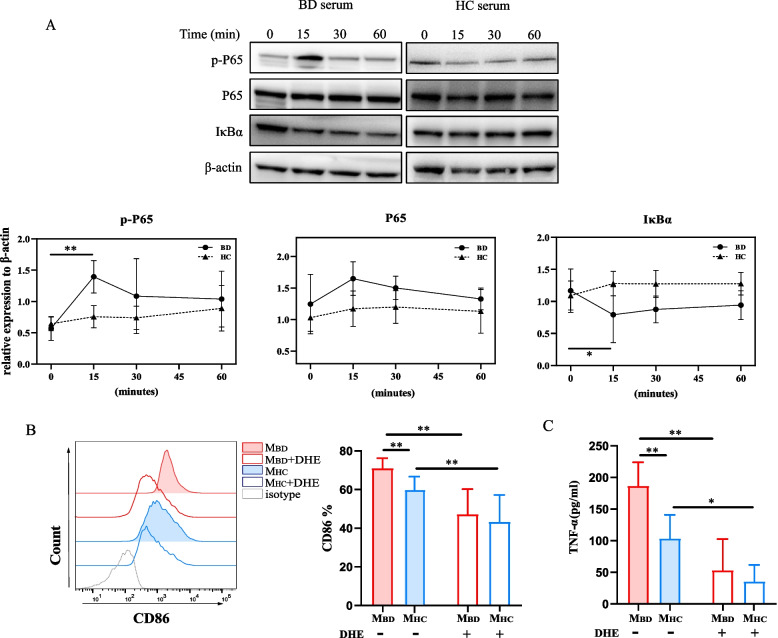
NF-κB pathway mediated BD serum-treated M1-like macrophage polarization. **A** Representative western blot images (upper) and summary (lower) of NF-κB p65, phospho-p65 and IκBα of macrophages treated with BD (*n*=3) serum or HC (*n*=3) serum. Macrophages were pretreated with DHE and then were stimulated with BD serum for 48 h. **B** Representative histograms (left) and summary (right) of CD86 expression on DHE-treated and untreated macrophages stimulated with BD (*n*=6) serum and HC (*n*=6) serum. **C** TNF-α production by DHE-treated and untreated macrophages stimulated with BD serum (*n*=6) and HC (*n*=6) serum. Data were shown as mean±SD. *, *p*<0.05; **, *p*<0.01, ***, *p*<0.001, ****, *p*<0.001 by two-way ANOVA. M_BD_, BD serum- treated macrophages; M_HC_, HC serum- treated macrophages

We further explored the underlying molecular mechanism of M1 macrophage polarization promoted by BD serum. Given that NF-κB pathways were enriched in transcriptome analysis (Fig. 3D–F) and participated in M1 macrophage polarization by TLRs [26], we examined NF-κB pathways in BD serum- and HC serum-treated macrophages. BD serum, but not HC serum, induced a significantly higher level of phosphorylated p65 (p-p65) (1.4 ± 0.3 vs 0.6 ± 0.2, *p*<0.05) and lower level of IκBα (0.8 ± 0.4 vs 1.2 ± 0.3, *p*<0.05) in macrophages (Fig. 4A) at 15 min. DHE, a specific NF-κB inhibitor, attenuated CD86 expression (71.7 ± 5.5% vs. 56.8 ± 4. 7%, *p*<0.001, Fig. 4B; Supplemental Figure S8) and TNF-α production (186.8 ± 37.2 vs 53.5 ± 49.1 pg/ml, *p*<0.0001, Fig. 4C) induced by BD serum. Additionally, higher levels of phosphorylated-JAK1 (1.0 ± 0.3 vs 0.5 ± 0.2, *p*<0.05) and phosphorylated-STAT1 (0.6 ± 0.2 vs 0.02 ± 0.02, *p*<0.0001) were observed in BD serum-treated macrophages (Supplemental Figure S9) at 30 min, which provided a new therapeutic mechanism of JAK inhibitor tofacitinib in BD [27]. Therefore, our data supported that the NF-κB pathway was implicated in the M1-like polarization of BD serum-treated macrophages.

## Discussion

This study demonstrated the M1 polarization of macrophages induced by BD serum, with enhanced ability of phagocytosis and facilitating Th1 differentiation. We found NF-κB pathway activation through transcriptome analysis and confirmed its regulation on BD serum-stimulated M1 polarization.

Imbalanced M1/M2 macrophage polarization has been reported in many autoimmune diseases, including SLE, RA, and IBD [7, 8]. M1 phenotype and M1/M2 ratio are increased in an HSV-induced BD mouse model, and IL-4 treatment reduces the M1/M2 ratio and ameliorates the disease [11]. Alpsoy et al. report that M1 macrophage markers CD11c and CD64 are strongly expressed in macrophages maintained in BD serum [12]. We observed an M1-like profile in BD serum-treated macrophages with elevated CD86, IL-12, and TNF-α and reduced CD163, and an M0-like profile in HC serum-treated HMDMs. M1 macrophages are induced by LPS, IFNγ and (or) TNF-α, while M2 macrophages are induced by IL4, IL-10, or IL-13 [6]. We and other investigators [28–30] have confirmed that the levels of IFNγ and TNF-α, but not IL-10, were higher in BD serum. However, neutralizing IFNγ and (or) TNF-α did not abrogate M1 polarization induced by BD serum (data not shown). In addition, serum levels of LPS in BD patients are positively correlated with mucous disease activity [31], and the expression of LPS receptor TLR4 is higher in BD macrophages [32]. We speculated that higher levels of LPS and IFNγ or TNF-α in BD serum might orchestrate to induce M1-like polarization, which deserves to explore in future studies. Nevertheless, our study and others indicate that M1 macrophage polarization is a key mechanism of BD, and targeting M1 macrophages might be a therapeutic approach for BD.

M1 macrophages exhibit higher phagocytic activity than M0 macrophages [33]. Accordingly, we found BD serum-stimulated macrophages showed enhanced phagocytotic ability through functional experiments and transcriptome analysis. Moreover, macrophages play an important role in the activation of the acquired immune response. In a chronic inflammatory context, M1 predominance and M2 insufficiency favor differentiation of T cell activation and differentiation [34, 35]. IL-12 and IL-23 and (or) IL-1β, produced by activated macrophages, induce Th1 and Th17 differentiation, respectively [36, 37]. IFNγ-producing Th1 cells and IL-17-producing Th17 cells are pathogenic in BD [10, 38, 39]. In this study, BD serum-treated macrophages upregulated IL-12 expression and promoted IFNγ^+^T-bet^+^ and IL-17A^+^RORγt^+^ CD4^+^T cell differentiation, suggesting that BD serum-induced M1-like macrophages contribute to the inflammation in BD via promotion of Th1 and Th17 differentiation.

Transcriptome data revealed DEGs related to chemotaxis, including CXCL1-3, CXCL5, and CCL13 in BD serum-treated macrophages. CXCL1-3 and CXCL5 are expressed by M1 macrophages and control the recruitment of neutrophils during tissue inflammation [40–42], and BD is a systemic vasculitis featured by notable neutrophil infiltration [43], CXCL1-3 and CXCL5 might be the key chemokines produced by BD macrophages to over-attract neutrophils. Additionally, CCL13 attracts monocytes, macrophages, and T cells [44, 45]. These chemokine genes suggest the M1-like BD serum-induced macrophages promote chemotactic activity to other immune cells and play a role in the pathogenesis of BD.

We found that NF-κB signaling regulated M1 macrophage polarization induced by BD serum. NF-κB is an important prototypic signaling cascade that drives classical (M1) activation of macrophages [46]. After stimulation, IκBα is phosphorylated, ubiquitinated, and degraded in the cytosol via activated IKK. Subsequently, NF-κB transcription factors were translocated into the nucleus to initiate downstream effector mechanisms and induction of proinflammatory mediators such as TNF-α and IL-6 [47]. In addition, NF-κB is a central mediator of priming signal for nod-like receptor (NLR) inflammasome [48], which pathway was also enriched in our transcriptomic analysis. In the study, we observed the degradation of IκBα and phosphorylation of p65, as well as overexpression of TNF-α in BD serum-treated macrophages, which suggested NF-κB signaling activation. NF-κB inhibitor attenuated M1 polarization of macrophages stimulated by BD serum. NF-κB activation could be induced by and, in turn, result in an amplified inflammatory cytokine profile in BD, including TNF-α, IL-1β, IL-6, IL-8, and IL-12 [10, 49–52], the combined effect of which provides M1 polarization environment.

Glucocorticoids, which inhibit NF-κB signaling through induction of IκBα synthesis and inhibition of NF-κB activity, are effective for BD [53]. We inferred that glucocorticoids may suppress inflammation by inhibiting M1 macrophage polarization in BD patients. Thus, our study added a new layer of treatment mechanism of glucocorticoids, and targeting the NF-κB pathway might be a potential approach to reduce the inflammatory response of BD.

## Conclusion

In summary, BD serum skews macrophage polarization toward the M1 phenotype by activating the NF-κB pathway, which shows enhanced phagocytosis and drives Th1 cell differentiation. Targeting abnormally polarized macrophages may be a potential therapeutic approach for BD.

## Supplementary Information

**Additional file 1: Supplemental Table S1.** Demographic and Clinical Characteristics of BD Patients and Healthy Controls.

**Additional file 2: Supplemental Table S2.** List of differential expression genes of BD serum- and HC serum-treated macrophages.

**Additional file 3: Supplemental Figure S1.** Representative FACS plot depicts the purity of monocytes, macrophages and naïve T CD4^+^T cells. **Supplemental Figure S2.** MFI analysis of BD serum-promoted macrophage polarization. **Supplemental Figure S3.** BD serum promotes CD86^+^CD163^-^CD206^-^ M1-like macrophage polarization. **Supplemental Figure S4.** BD serum-treated macrophages facilitate Th1 differentiation under Th0 condition. **Supplemental Figure S5.** MFI analysis of T-bet in BD serum-treated macrophages. **Supplemental Figure S6**. BD serum-treated macrophages facilitate Th17 differentiation. **Supplemental Figure S7.** BD serum-treated macrophages produced more CXCL2 and CXCL3. **Supplemental Figure S8.** NF-κB inhibition attenuated CD86 expression on macrophages stimulated by BD serum. **Supplemental Figure S9.** Activated JAK/STAT pathway in BD serum-treated macrophages.

## Data Availability

The original contributions presented in the study are included in the article/Supplementary Materials. The dataset supporting the conclusions of this article is available in the Gene Expression Omnibus (GEO) database (accession: GSE185919). Further inquiries can be directed to the corresponding authors.

## Abbreviations

BD: Behçet’s disease
HC: Healthy controls
HMDMs: Human monocyte-derived macrophages
LPS: Lipopolysaccharides
IFNγ: Interferon-γ
SLE: Systemic lupus erythematosus
RA: Rheumatoid arthritis
SS: Sjögren’s syndrome
IBD: Inflammatory bowel diseases
PBMCs: Peripheral blood mononuclear cells
GO: Gene Ontology
KEGG: Kyoto Encyclopedia of Genes and Genomes
