# Correction: Macrophage polarization toward M1 phenotype through NF-κB signaling in patients with Behcet’s disease

**DOI:** 10.1186/s13075-022-02962-z

**Published:** 2022-12-16

**Authors:** Xiuhua Wu, Zhimian Wang, Jing Shi, Xin Yu, Chaoran Li, Jinjing Liu, Fengchun Zhang, Hua Chen, Wenjie Zheng

**Affiliations:** 1 Department of Rheumatology and Clinical Immunology, Chinese Academy of Medical Sciences & Peking Union Medical College; National Clinical Research Center for Dermatologic and Immunologic Diseases (NCRC-DID), Ministry of Science & Technology; State Key Laboratory of Complex Severe and Rare Diseases, Peking Union Medical College Hospital (PUMCH); Key Laboratory of Rheumatology and Clinical Immunology, Ministry of Education, Beijing, 100730 China; 2 Department of Rheumatology and Immunology, Tianjin Medical University General Hospital, Tianjin, 300052 China; 3 Department of Rheumatology, Zhongshan Hospital, Fudan University, Shanghai, 200032 China

**Correction: Arthritis Res Ther 24, 249 (2022)**

**https://doi.org/10.1186/s13075-022-02938-z**

Following publication of the original article [1], the authors have identified an error in Figs. 1, 2, 3 and 4. The correct figures are given below.

**Fig. 1:**
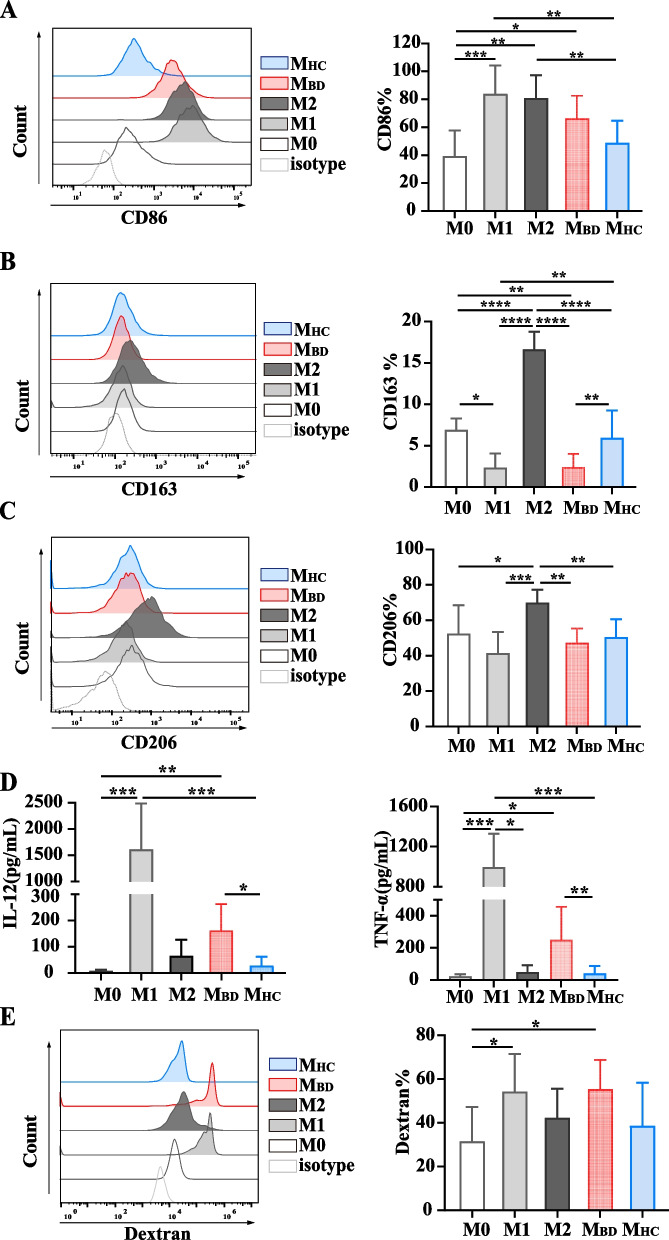
BD serum promotes M1-like macrophage polarization. Resting macrophages (M0) were stimulated with M1 condition (100ng/ml LPS+ 20ng/ml IFNγ), M2 condition (20ng/ml IL-4+ 20ng/ml IL-13), BD serum or HC serum for 48 h. **A–C** Representative histograms (left) and summary (right) of CD86, CD163 and CD206 expression level of macrophages stimulated with M0 (*n*=6), M1 (*n*=6), and M2 (*n*=6) conditions, as well as BD (*n*=12) serum and HC (*n*=12) serum. Data were expressed as mean±SD and were analyzed using one-way ANOVA. **D** IL-12 and TNF-α production by macrophages stimulated with M0 (*n*=6), M1 (*n*=6), and M2 (*n*=6) conditions, as well as BD (*n*=12) serum and HC (*n*=12) serum. Data were expressed as mean±SD and were analyzed using Kruskal-Wallis test. **E** Representative histograms (left) and summary (right) of dextran uptake by macrophages stimulated with M0 (*n*=7), M1 (*n*=7), M2 (*n*=7) conditions, and BD (*n*=9) serum and HC (*n*=9) serum. Data were expressed as mean±SD and were analyzed using one-way ANOVA. *, *p*<0.05; **, *p*<0.01; ***, *p*<0.001, ****, *p*<0.001. M_BD_, BD serum-treated macrophages; M_HC_, HC serum-treated macrophages

**Fig. 2:**
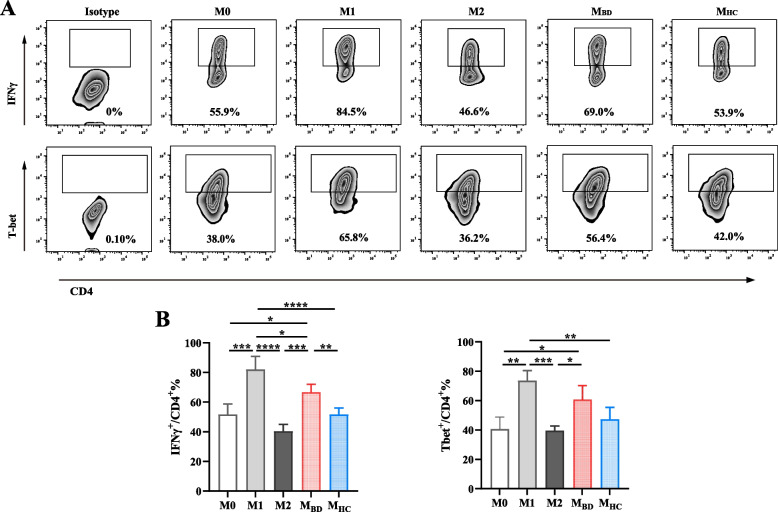
BD serum-treated macrophages facilitate Th1 differentiation. Naive CD4+ T cells were incubated with M0, M1, M2, BD serum- and HC serum-treated macrophages in Th1 condition (5μg/ml anti-CD3, 5μg/ml anti-CD28, 5μg/ml anti-IL-4, and 10ng/ml IL-2) for 5 days. **A** Representative flow cytometry plots and **B** summary of IFNγ and T-bet [*n*(M0)=3, *n*(M1)=3, *n*(M2)=3, *n*(M_BD_)=5, *n*(M_HC_)=5] expression levels in CD4+ T cells. Data were shown as mean±SD. *, *p*<0.05; **, *p*<0.01; ***, *p*<0.001, ****, *p*<0.001 by one-way ANOVA. M_BD_, BD serum- treated macrophages; M_HC_, HC serum- treated macrophages

**Fig. 3:**
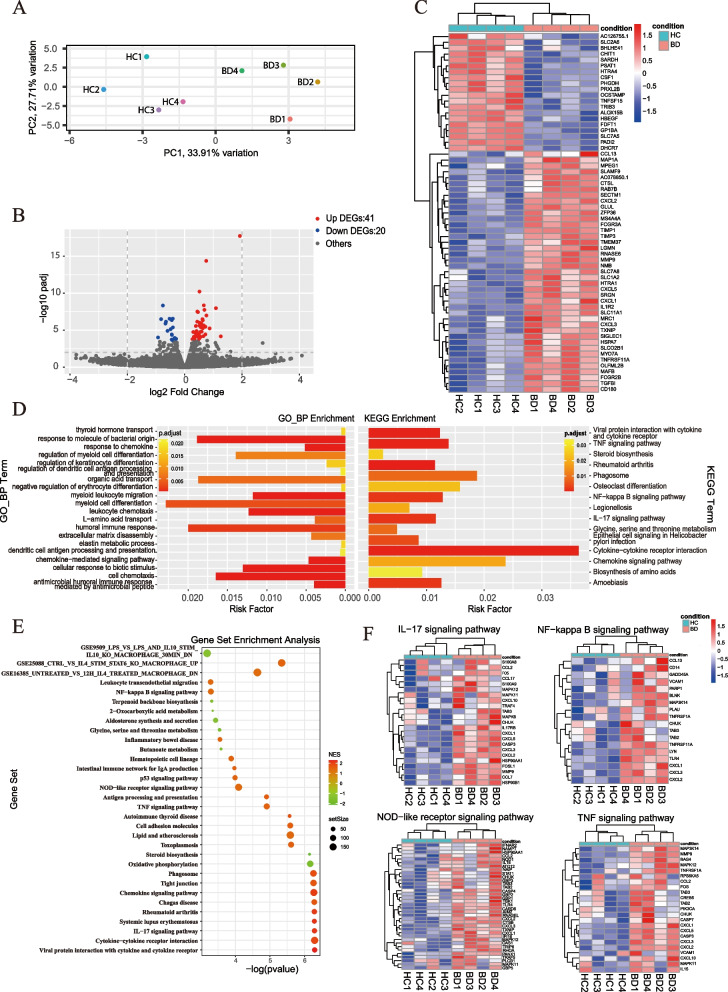
Transcriptome analysis of BD serum- and HC serum-treated macrophages. HMDMs were stimulated with serum from four treatment-naïve active BD patients and matched healthy volunteers for 48 h, and total RNA was extracted for RNA-seq analysis. **A** Principal component analysis (PCA) of BD serum-treated and HC serum-treated macrophages. **B** Volcano plot of upregulated (red, *n*=41) and downregulated (blue, *n*=20) DEGs in BD serum-treated macrophages compared with HC serum-treated macrophages. **C** Heatmap of DEGs between BD serum- and HC serum-treated macrophages. **D** GO biological process enrichment analysis and KEGG enrichment analysis between BD serum- and HC serum-treated macrophage. **E**, **F** Dot plots (left) showed Gene Set Enrichment Analysis (GSEA) of BD serum- and HC serum-treated macrophage. Representative enriched gene sets were illustrated by heatmap (right). DEGs, differentially expressed genes; GO, gene ontology; KEGG, Kyoto Encyclopedia of Genes and Genomes

**Fig. 4:**
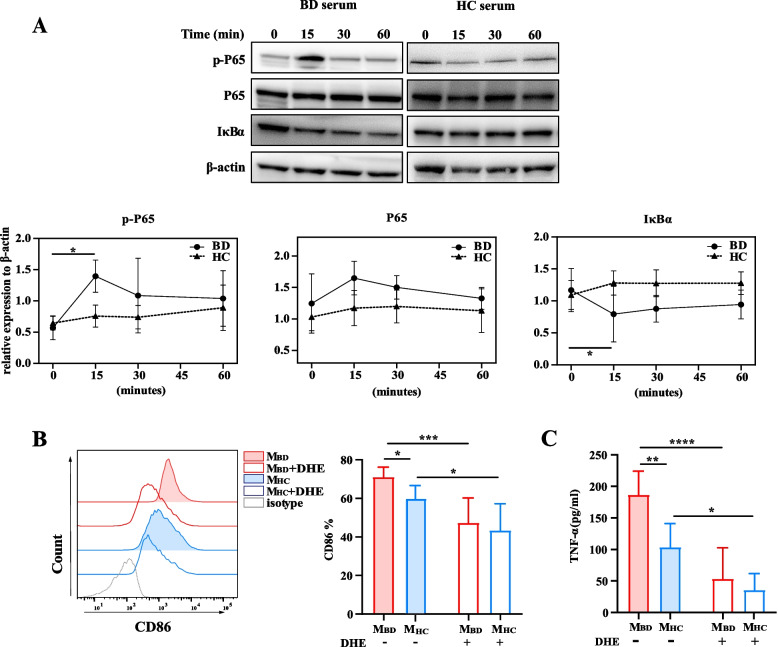
NF-κB pathway mediated BD serum-treated M1-like macrophage polarization. **A** Representative western blot images (upper) and summary (lower) of NF-κB p65, phospho-p65 and IκBα of macrophages treated with BD (*n*=3) serum or HC (*n*=3) serum. Macrophages were pretreated with DHE and then were stimulated with BD serum for 48 h. **B** Representative histograms (left) and summary (right) of CD86 expression on DHE-treated and untreated macrophages stimulated with BD (*n*=6) serum and HC (*n*=6) serum. **C** TNF-α production by DHE-treated and untreated macrophages stimulated with BD serum (*n*=6) and HC (*n*=6) serum. Data were shown as mean±SD. *, *p*<0.05; **, *p*<0.01, ***, *p*<0.001, ****, *p*<0.001 by two-way ANOVA. M_BD_, BD serum- treated macrophages; M_HC_, HC serum- treated macrophages

The original article [1] has been updated.
