# Supplementary material for: Macrophage polarization toward M1 phenotype through NF-κB signaling in patients with Behçet’s disease
Source: Arthritis Res Ther. 2022 Nov 4;24:249. doi: 10.1186/s13075-022-02938-z (PMC9635113; doi:10.1186/s13075-022-02938-z)
Supplement: Supplementary file 1 — Additional file 1: Supplemental Table S1. Demographic and Clinical Characteristics of BD Patients and Healthy Controls. [file 13075_2022_2938_MOESM1_ESM.docx]

***Supplemental*** ***Table S1***

- 1. **Demographic and Clinical Characteristics of BD Patients and Healthy Controls***

| Feature | BD  (n=45) | Healthy controls  (n=45) |
| --- | --- | --- |
| Gender, Male (n, %)  Age, mean±SD, years | 27 (60.0)  33.0±11.2 | 27(60.0)  35.6±13.2 |
| Disease duration, mean±SD, months | 94.4±78.4 | / |
| BDCAF 2006, median (range) | 2 (0-5) | / |
| Manifestations (n, %) | | / |
| Recurrent oral ulcers | 45 (100) |  |
| Genital ulcers | 32 (71.1) |  |
| Skin lesions | 29 (64.5) |  |
| Ocular involvement | 5 (11.1) |  |
| Pathergy test positive | 8 (17.8) |  |
| Vascular involvement | 15 (33.3) |  |
| Gastrointestinal involvement | 7 (15.6) |  |
| Central nervous system involvement | 3 (6.7) |  |
| Cardiac involvement | 9 (20.0) |  |
| ESR, median (range), mm/hour | 36 (2-140) | / |
| CRP, median (range), mg/L | 33.8 (0.2-226.0) / | |

Data are expressed as number (percentage) or median (range).

Abbreviation: BDCAF 2006: BD Current Activity Form 2006. CRP: C reactive protein. ESR: erythrocyte sedimentation rate.

* Forty-five matched BD patients and HC were involved in different parts of the project, including phenotypic, functional, and mechanism studies.

**1.2 Clinical characteristics of BD patients used for phenotype analysis (n=12)**

| **Patient** | **Gender** | **ESR (****mm/h)** | **hsCRP (****mg/L)** | **BDCAF 2006** | **Clinical manifestations** |
| --- | --- | --- | --- | --- | --- |
|  |  |  |  |  |  |
| **1** | M | 39 | 23.04 | 1 | Oral and genital ulcers, Vascular manifestations |
| **2** | M | 77 | 104.68 | 2 | Oral ulcer, Skin lesions, Positive pathergy test |
| **3** | F | 35 | 13.87 | 2 | Oral and genital ulcers |
| **4** | M | 20 | 30.4 | 5 | Oral and genital ulcers, Intestinal ulcer |
| **5** | F | 34 | 9.84 | 2 | Oral and genital ulcers, Skin lesions |
| **6** | M | 80 | 62.86 | 2 | Oral ulcer, Skin lesions, Positive pathergy test |
| **7** | F | 33 | 33.77 | 1 | Oral and genital ulcers, Positive pathergy test |
| **8** | M | 11 | 20.79 | 1 | Oral ulcer, Skin lesions, Vascular manifestations |
| **9** | M | 44 | 21.71 | 3 | Oral and genital ulcers, Positive pathergy test, Uveitis |
| **10** | M | 30 | 5.86 | 1 | Oral and genital ulcers, Skin lesions |
| **11** | F | 54 | 40.26 | 4 | Oral and genital ulcers, Neurological manifestations |
| **12** | M | 78 | 226.01 | 1 | Oral and genital ulcers, Skin lesions, Uveitis |

Abbreviation: M, Male. F, Female. BDCAF 2006: BD Current Activity Form 2006. CRP: C reactive protein. ESR: erythrocyte sedimentation rate.
