# Supplementary material for: Macrophage polarization toward M1 phenotype through NF-κB signaling in patients with Behçet’s disease
Source: Arthritis Res Ther. 2022 Nov 4;24:249. doi: 10.1186/s13075-022-02938-z (PMC9635113; doi:10.1186/s13075-022-02938-z)
Supplement: Supplementary file 2 — Additional file 2: Supplemental Table S2. List of differential expression genes of BD serum- and HC serum-treated macrophages. [file 13075_2022_2938_MOESM2_ESM.docx]

***Supplemental*** ***Table S2***

|  | **Supplementary Table S2. List of differential expression genes of BD serum- and HC serum-stimulated macrophages.** | | | | | | | | |
| --- | --- | --- | --- | --- | --- | --- | --- | --- | --- |
|  | Gene | Base Mean | log2 Fold Change | lfc SE | Stat | P value | P adj | Ensembl Gene ID | Entrez Gene ID |
| 1 | CXCL5 | 161.02046 | 1.92562407 | 0.219596862 | -8.76891 | 1.80E-18 | 2.59E-14 | ENSG00000163735 | 6374 |
| 2 | CCL13 | 110.78476 | 1.26012437 | 0.316145924 | -3.98589 | 6.72E-05 | 0.021341 | ENSG00000181374 | 6357 |
| 3 | IL1R2 | 192.71773 | 1.089584555 | 0.190345279 | -5.72425 | 1.04E-08 | 2.48E-05 | ENSG00000115590 | 7850 |
| 4 | CXCL1 | 213.22322 | 0.870116966 | 0.191740616 | -4.53799 | 5.68E-06 | 0.002807 | ENSG00000163739 | 2919 |
| 5 | OLFML2B | 898.68972 | 0.758321111 | 0.142431511 | -5.32411 | 1.01E-07 | 0.000145 | ENSG00000162745 | 25903 |
| 6 | FCGR3A | 6435.1526 | 0.751784663 | 0.095794104 | -7.84792 | 4.23E-15 | 3.03E-11 | ENSG00000203747 | 2214 |
| 7 | NA | 269.22444 | 0.724265331 | 0.155792439 | -4.64891 | 3.34E-06 | 0.001913 | ENSG00000248187 | - |
| 8 | TMEM37 | 343.83455 | 0.721804406 | 0.174854168 | -4.12804 | 3.66E-05 | 0.01333 | ENSG00000171227 | 140738 |
| 9 | SIGLEC1 | 2278.6943 | 0.688259029 | 0.122196379 | -5.6324 | 1.78E-08 | 3.64E-05 | ENSG00000088827 | 6614 |
| 10 | TGFBI | 13349.057 | 0.667323199 | 0.113652376 | -5.87162 | 4.32E-09 | 1.30E-05 | ENSG00000120708 | 7045 |
| 11 | CD180 | 558.70783 | 0.66176703 | 0.13985721 | -4.73173 | 2.23E-06 | 0.00145 | ENSG00000134061 | 4064 |
| 12 | SLC1A2 | 497.51275 | 0.652870607 | 0.163199508 | -4.00044 | 6.32E-05 | 0.020597 | ENSG00000110436 | 6506 |
| 13 | FCGR2B | 877.7676 | 0.635159191 | 0.13945771 | -4.55449 | 5.25E-06 | 0.002688 | ENSG00000072694 | 2213 |
| 14 | NA | 1578.8613 | 0.632055834 | 0.125339195 | -5.04276 | 4.59E-07 | 0.000411 | ENSG00000225217 | - |
| 15 | SLAMF9 | 910.00959 | 0.621059549 | 0.12146165 | -5.11322 | 3.17E-07 | 0.000324 | ENSG00000162723 | 89886 |
| 16 | SECTM1 | 541.91078 | 0.592410049 | 0.137098408 | -4.32106 | 1.55E-05 | 0.006745 | ENSG00000141574 | 6398 |
| 17 | HTRA1 | 712.73587 | 0.576521553 | 0.118999638 | -4.84473 | 1.27E-06 | 0.000867 | ENSG00000166033 | 5654 |
| 18 | MYO7A | 1005.3697 | 0.567008483 | 0.123117976 | -4.60541 | 4.12E-06 | 0.002215 | ENSG00000137474 | 4647 |
| 19 | NMB | 287.38947 | 0.557344926 | 0.141184124 | -3.94765 | 7.89E-05 | 0.023569 | ENSG00000197696 | 4828 |
| 20 | CXCL3 | 758.0664 | 0.544065418 | 0.126475795 | -4.30174 | 1.69E-05 | 0.007133 | ENSG00000163734 | 2921 |
| 21 | CXCL2 | 1057.8063 | 0.521515912 | 0.106449509 | -4.89919 | 9.62E-07 | 0.000726 | ENSG00000081041 | 2920 |
| 22 | SLCO2B1 | 9337.914 | 0.517131633 | 0.109643649 | -4.71648 | 2.40E-06 | 0.001496 | ENSG00000137491 | 11309 |
| 23 | TIMP1 | 6099.6198 | 0.516693103 | 0.078948522 | -6.54468 | 5.96E-11 | 2.85E-07 | ENSG00000102265 | 7076 |
| 24 | TNFRSF11A | 416.53017 | 0.506103824 | 0.132943768 | -3.8069 | 0.000141 | 0.038058 | ENSG00000141655 | 8792 |
| 25 | MAP1A | 605.35197 | 0.501570613 | 0.120089512 | -4.17664 | 2.96E-05 | 0.01116 | ENSG00000166963 | 4130 |
| 26 | SRGN | 8417.428 | 0.463114269 | 0.083996197 | -5.51351 | 3.52E-08 | 5.60E-05 | ENSG00000122862 | 5552 |
| 27 | MPEG1 | 4515.8428 | 0.446854438 | 0.112233202 | -3.98148 | 6.85E-05 | 0.021341 | ENSG00000197629 | 219972 |
| 28 | MS4A4A | 2718.893 | 0.441398044 | 0.07962198 | -5.54367 | 2.96E-08 | 5.31E-05 | ENSG00000110079 | 51338 |
| 29 | MAFB | 4861.8536 | 0.438245731 | 0.088297458 | -4.96329 | 6.93E-07 | 0.000558 | ENSG00000204103 | 9935 |
| 30 | RAB7B | 3458.9004 | 0.417722302 | 0.099468942 | -4.19952 | 2.67E-05 | 0.010362 | ENSG00000276600 | 338382 |
| 31 | MRC1 | 13102.872 | 0.416267562 | 0.102228707 | -4.07192 | 4.66E-05 | 0.015913 | ENSG00000260314 | 4360 |
| 32 | CTSL | 20252.739 | 0.413891359 | 0.094930158 | -4.35996 | 1.30E-05 | 0.005827 | ENSG00000135047 | 1514 |
| 33 | TIMP3 | 10198.961 | 0.389381378 | 0.082983912 | -4.69225 | 2.70E-06 | 0.001614 | ENSG00000100234 | 7078 |
| 34 | SLC7A8 | 3073.5012 | 0.378263829 | 0.09400626 | -4.02382 | 5.73E-05 | 0.019088 | ENSG00000092068 | 23428 |
| 35 | LGMN | 7266.9649 | 0.365235851 | 0.085653996 | -4.26408 | 2.01E-05 | 0.007992 | ENSG00000100600 | 5641 |
| 36 | RNASE6 | 1814.4357 | 0.346733416 | 0.091427375 | -3.79245 | 0.000149 | 0.038877 | ENSG00000169413 | 6039 |
| 37 | TXNIP | 9479.8667 | 0.324195379 | 0.086512624 | -3.74738 | 0.000179 | 0.044162 | ENSG00000265972 | 10628 |
| 38 | SLC11A1 | 4884.0495 | 0.320926804 | 0.081082615 | -3.95802 | 7.56E-05 | 0.023048 | ENSG00000018280 | 6556 |
| 39 | ZFP36 | 2616.4579 | 0.316238568 | 0.083686491 | -3.77885 | 0.000158 | 0.040329 | ENSG00000128016 | 7538 |
| 40 | MMP9 | 301290.99 | 0.299640937 | 0.069754105 | -4.29567 | 1.74E-05 | 0.007133 | ENSG00000100985 | 4318 |
| 41 | GLUL | 37301.247 | 0.271355274 | 0.071930448 | -3.77247 | 0.000162 | 0.040648 | ENSG00000135821 | 2752 |
| 42 | FDFT1 | 2826.1684 | -0.300342259 | 0.079048923 | 3.799448 | 0.000145 | 0.038494 | ENSG00000079459 | 2222 |
| 43 | DHCR7 | 1602.598 | -0.348674553 | 0.090157356 | 3.8674 | 0.00011 | 0.031535 | ENSG00000172893 | 1717 |
| 44 | PRXL2B | 4429.1253 | -0.355291926 | 0.093162211 | 3.813691 | 0.000137 | 0.038058 | ENSG00000157870 | 127281 |
| 45 | HTRA4 | 1521.4821 | -0.363034595 | 0.095276672 | 3.81032 | 0.000139 | 0.038058 | ENSG00000169495 | 203100 |
| 46 | OCSTAMP | 1566.8029 | -0.369932147 | 0.099566922 | 3.715412 | 0.000203 | 0.048466 | ENSG00000149635 | 128506 |
| 47 | CSF1 | 10787.374 | -0.410995112 | 0.089296567 | 4.602586 | 4.17E-06 | 0.002215 | ENSG00000184371 | 1435 |
| 48 | CHIT1 | 34252.763 | -0.414828772 | 0.0806699 | 5.142299 | 2.71E-07 | 0.000324 | ENSG00000133063 | 1118 |
| 49 | SARDH | 595.84615 | -0.435460918 | 0.117477991 | 3.706745 | 0.00021 | 0.049333 | ENSG00000123453 | 1757 |
| 50 | HBEGF | 1265.9078 | -0.445743962 | 0.10892562 | 4.092187 | 4.27E-05 | 0.01494 | ENSG00000113070 | 1839 |
| 51 | ALOX15B | 893.71346 | -0.450413201 | 0.109211422 | 4.124232 | 3.72E-05 | 0.01333 | ENSG00000179593 | 247 |
| 52 | SLC7A5 | 1467.7322 | -0.450707793 | 0.089158732 | 5.055117 | 4.30E-07 | 0.000411 | ENSG00000103257 | 8140 |
| 53 | BHLHE41 | 3255.8794 | -0.452294897 | 0.121029171 | 3.737073 | 0.000186 | 0.045231 | ENSG00000123095 | 79365 |
| 54 | SLC2A6 | 1910.8569 | -0.487927665 | 0.108642942 | 4.491112 | 7.09E-06 | 0.003385 | ENSG00000160326 | 11182 |
| 55 | PADI2 | 778.38831 | -0.527136576 | 0.102622945 | 5.136635 | 2.80E-07 | 0.000324 | ENSG00000117115 | 11240 |
| 56 | PHGDH | 626.63824 | -0.598922941 | 0.136953177 | 4.373195 | 1.22E-05 | 0.005662 | ENSG00000092621 | 26227 |
| 57 | PSAT1 | 689.14353 | -0.653550188 | 0.131733051 | 4.961171 | 7.01E-07 | 0.000558 | ENSG00000135069 | 29968 |
| 58 | TRIB3 | 478.60922 | -0.669378744 | 0.138178683 | 4.844298 | 1.27E-06 | 0.000867 | ENSG00000101255 | 57761 |
| 59 | GP1BA | 483.07134 | -0.758870409 | 0.129427199 | 5.863299 | 4.54E-09 | 1.30E-05 | ENSG00000185245 | 2811 |
| 60 | TNFSF15 | 366.1522 | -0.82289111 | 0.16090814 | 5.114043 | 3.15E-07 | 0.000324 | ENSG00000181634 | 9966 |
| 61 | NA | 143.47609 | -0.929627831 | 0.238131872 | 3.903836 | 9.47E-05 | 0.027697 | ENSG00000205746 | - |
