# Supplementary material for: Macrophage polarization toward M1 phenotype through NF-κB signaling in patients with Behçet’s disease
Source: Arthritis Res Ther. 2022 Nov 4;24:249. doi: 10.1186/s13075-022-02938-z (PMC9635113; doi:10.1186/s13075-022-02938-z)
Supplement: Supplementary file 3 — Additional file 3: Supplemental Figure S1. Representative FACS plot depicts the purity of monocytes, macrophages and naïve T CD4+T cells. Supplemental Figure S2. MFI analysis of BD serum-promoted macrophage polarization. Supplemental Figure S3. BD serum promotes CD86+CD163-CD206- M1-like macrophage polarization. Supplemental Figure S4. BD serum-treated macrophages facilitate Th1 differentiation under Th0 condition. Supplemental Figure S5. MFI analysis of T-bet in BD serum-treated macrophages. Supplemental Figure S6. BD serum-treated macrophages facilitate Th17 differentiation. Supplemental Figure S7. BD serum-treated macrophages produced more CXCL2 and CXCL3. Supplemental Figure S8. NF-κB inhibition attenuated CD86 expression on macrophages stimulated by BD serum. Supplemental Figure S9. Activated JAK/STAT pathway in BD serum-treated macrophages. [file 13075_2022_2938_MOESM3_ESM.docx]

Supplemental Figures


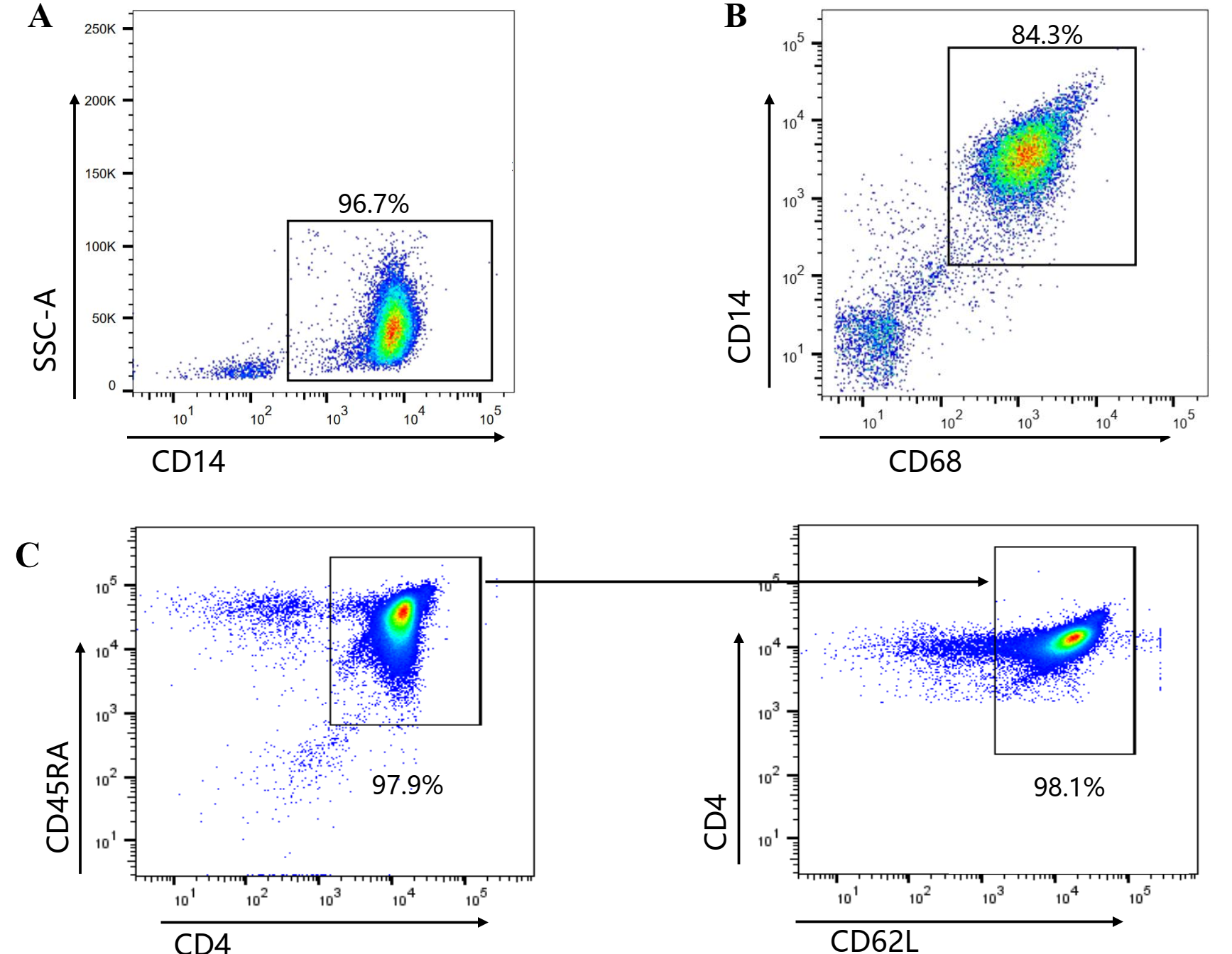


**Supplemental Figure S1. Representative FACS plot depicts the purity of monocytes, macrophages and naïve T CD4+T cells.**

1. The purity of monocytes isolated from PBMCs using CD14^+^ MicroBeads.
2. Monocytes were incubated in complete DMEM medium supplemented with 50ng/ml M-CSF for 7 days to differentiate into HMDMs. The purity of HMDMs was determined by flow cytometry.
3. The purity of naïve CD4^+^ T cells isolated from PBMCs using naive CD4^+^ T cell isolation kit II.


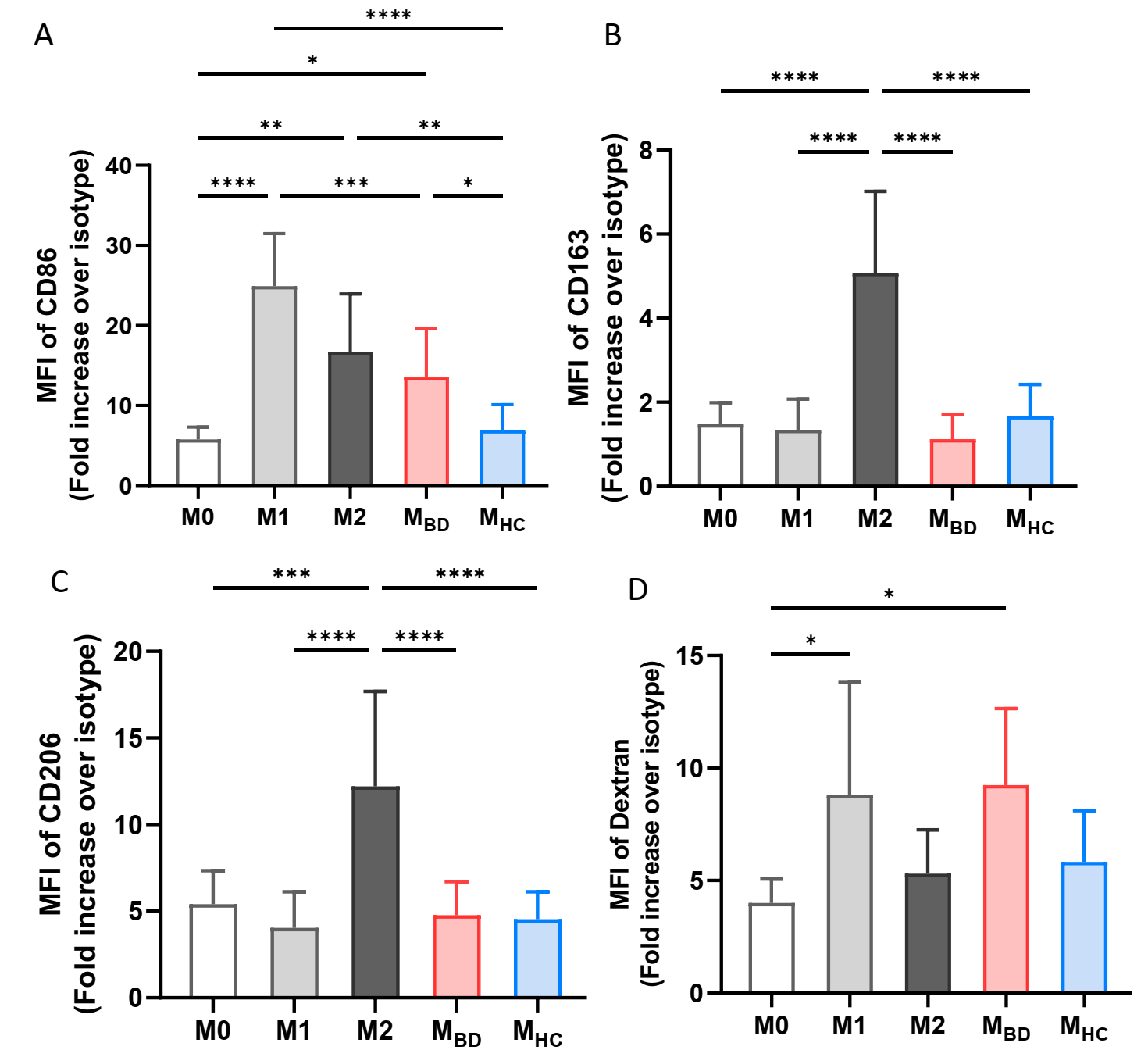


**Supplemental Figure S2. MFI analysis of BD serum-promoted macrophage polarization.**

Resting macrophages (M0) were stimulated with M1 condition (100ng/ml LPS+ 20ng/ml IFNγ), M2 condition (20ng/ml IL-4+ 20ng/ml IL-13), BD serum or HC serum for 48 hours. The MFIs of surface markers were determined by flow cytometry.

(A-C) Summary graph of CD86, CD163 and CD206 mean fluorescence intensity (MFI) level of macrophages stimulated with M0 (n=6), M1 (n=6) and M2 (n=6) conditions, as well as BD (n=12) serum and HC (n=12) serum.

(D) Summary graph of dextran MFI of macrophages stimulated with M0 (n=7), M1 (n=7), M2 (n=7) conditions, and BD (n=9) serum and HC (n=9) serum.

Data were expressed as mean±SD.*, p<0.05; **, p<0.01; ***, p<0.001, ****, p<0.001 by One-way ANOVA. M_BD_, BD serum-treated macrophages; M_HC_, HC serum-treated macrophages.


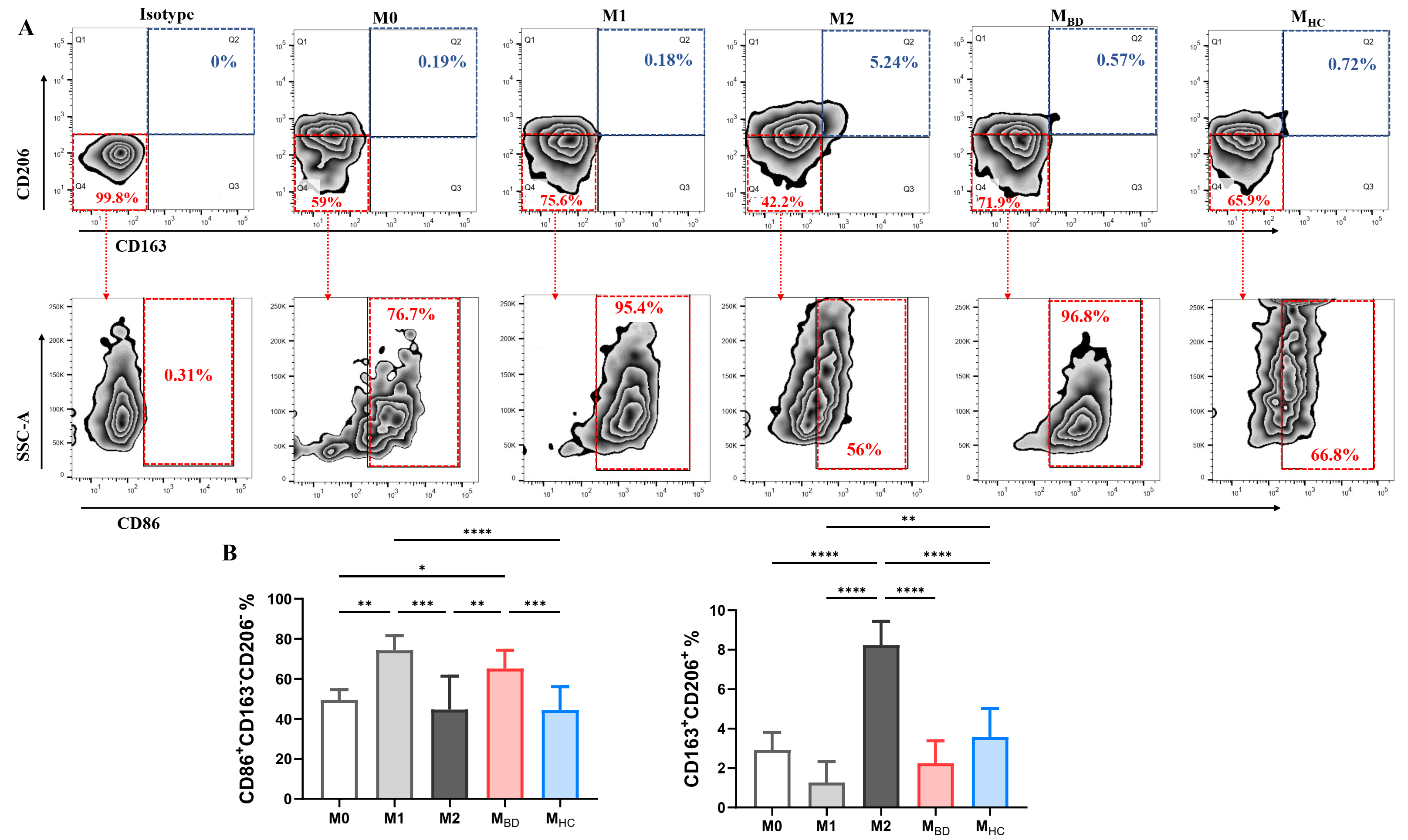


**Supplemental Figure S3.** **BD serum promotes CD86^+^CD163^-^CD206^-^ M1-like macrophage polarization.**

Resting macrophages (M0) were stimulated with M1 condition (100ng/ml LPS+ 20ng/ml IFNγ), M2 condition (20ng/ml IL-4+ 20ng/ml IL-13), BD serum or HC serum for 48 hours. CD86, CD163 and CD206 expression were determined by flow cytometry.

(A) Representative flow cytometry plots and (B) summary of the proportion of CD86^+^CD163^-^CD206^-^ M1 and CD163^+^CD206^+^ M2 macrophages in M0, M1 and M2 conditions (n=6), as well as BD serum- and HC serum-treated macrophages (n=12). Data was shown as the mean ± SD. One-way ANOVA was used to compare the differences between groups. *, p<0.05; **, p<0.01; ***, p<0.001; ****, p<0.0001.


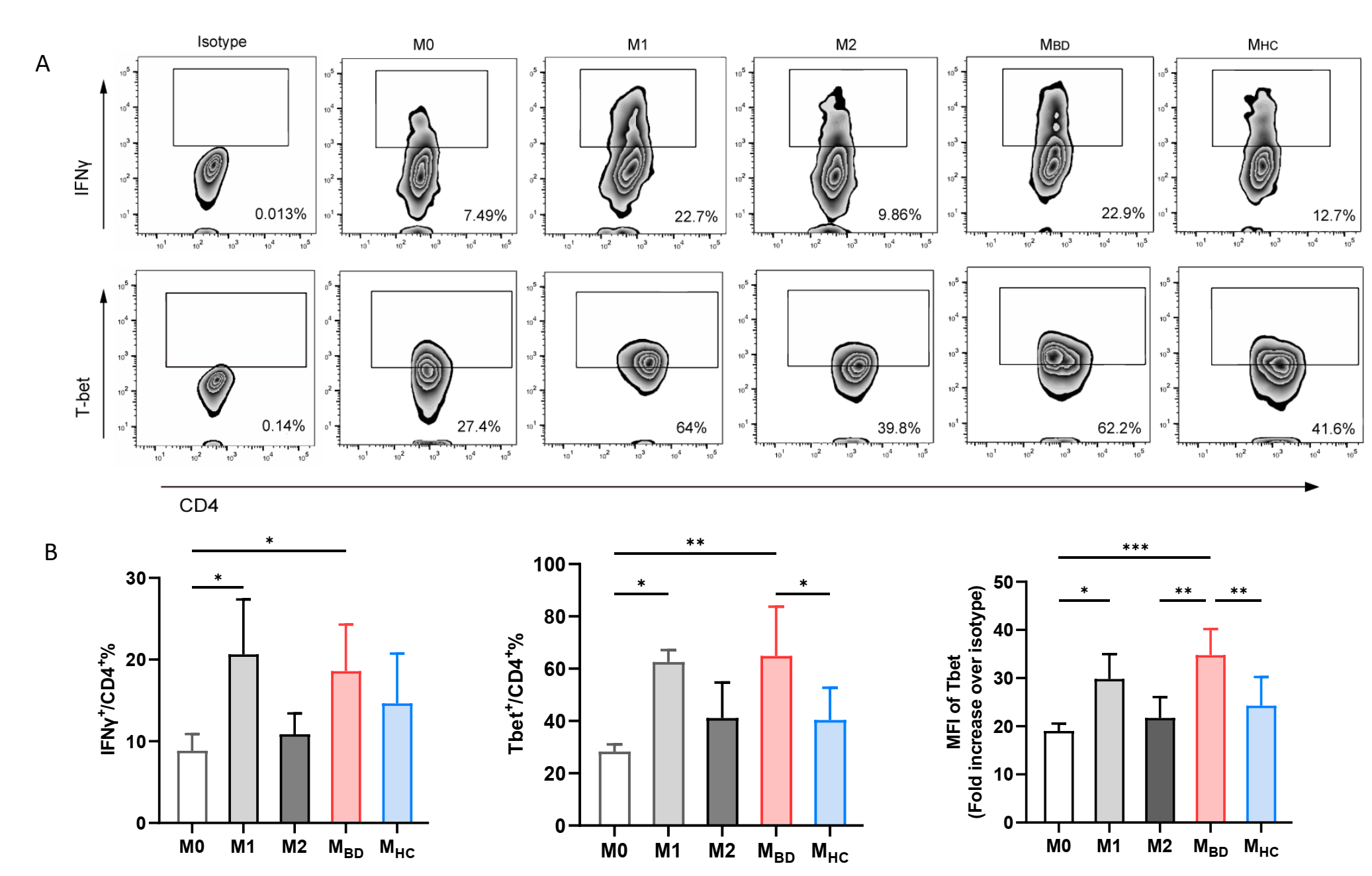


**Supplemental Figure S4. BD serum-treated macrophages facilitate Th1 differentiation under Th0 condition.**

Naive CD4^+^ T cells were incubated with M0, M1, M2, BD serum- and HC serum- treated macrophages in Th0 condition (5ug/ml anti-CD3, 5ug/ml anti-CD28, and 10ng/ml IL-2) for 5 days and were analyzed by flow cytometry.

(A) Representative flow cytometry plots and (B) summary of IFNγ, T-bet expression levels and T-bet MFI levels in CD4^+^ T cells. [n(M0)=4, n(M1)=4, n(M2)=4, n(M_BD_)=8, n(M_HC_)=8]

Data were shown as mean±SD. *, p<0.05; **, p<0.01; ***, p<0.001 by One-way ANOVA. M_BD_, BD serum- treated macrophages; M_HC_, HC serum- treated macrophages.

**Supplemental Figure S5. MFI analysis of T-bet in BD serum-treated macrophages.**

Naive CD4^+^ T cells were incubated with M0, M1, M2, BD serum- and HC serum- treated macrophages in Th1 condition (5ug/ml anti-CD3, 5ug/ml anti-CD28, 5ug/ml anti-IL-4 and 10ng/ml IL-2) for 5 days and harvested for flow cytometry analysis.

Summary graph of T-bet MFI levels in CD4^+^ T cells [n(M0)=3, n(M1)=3, n(M2)=3, n(M_BD_)=5, n(M_HC_)=5] were shown.

Data were shown as mean±SD. *, p<0.05; **, p<0.01 by One-way ANOVA. M_BD_, BD serum- treated macrophages; M_HC_, HC serum- treated macrophages.


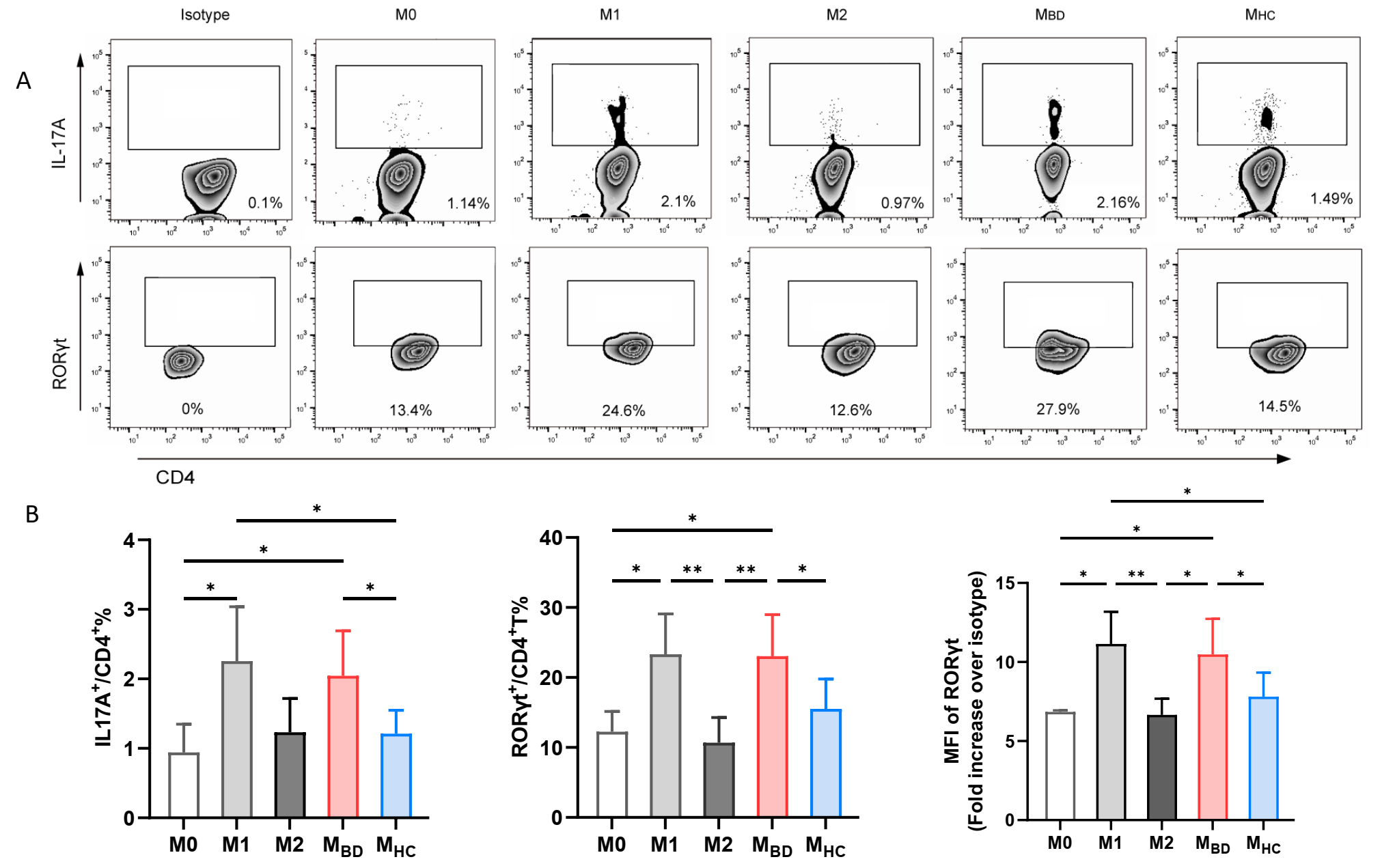


**Supplemental Figure S6. BD serum-treated macrophages facilitate Th17 differentiation.**

Naive CD4^+^ T cells were incubated with M0, M1, M2, BD serum- and HC serum- treated macrophages in Th0 condition (5ug/ml anti-CD3, 5ug/ml anti-CD28, and 10ng/ml IL-2) for 5 days and harvested for flow cytometry analysis.

(A) Representative flow cytometry plots and (B) summary of IL-17A, RORγt expression levels and RORγt MFI levels in CD4^+^ T cells. [n(M0)=4, n(M1)=4, n(M2)=4, n(M_BD_)=8, n(M_HC_)=8]

Data were shown as mean±SD. *, p<0.05; **, p<0.01 by One-way ANOVA. M_BD_, BD serum- treated macrophages; M_HC_, HC serum- treated macrophages.


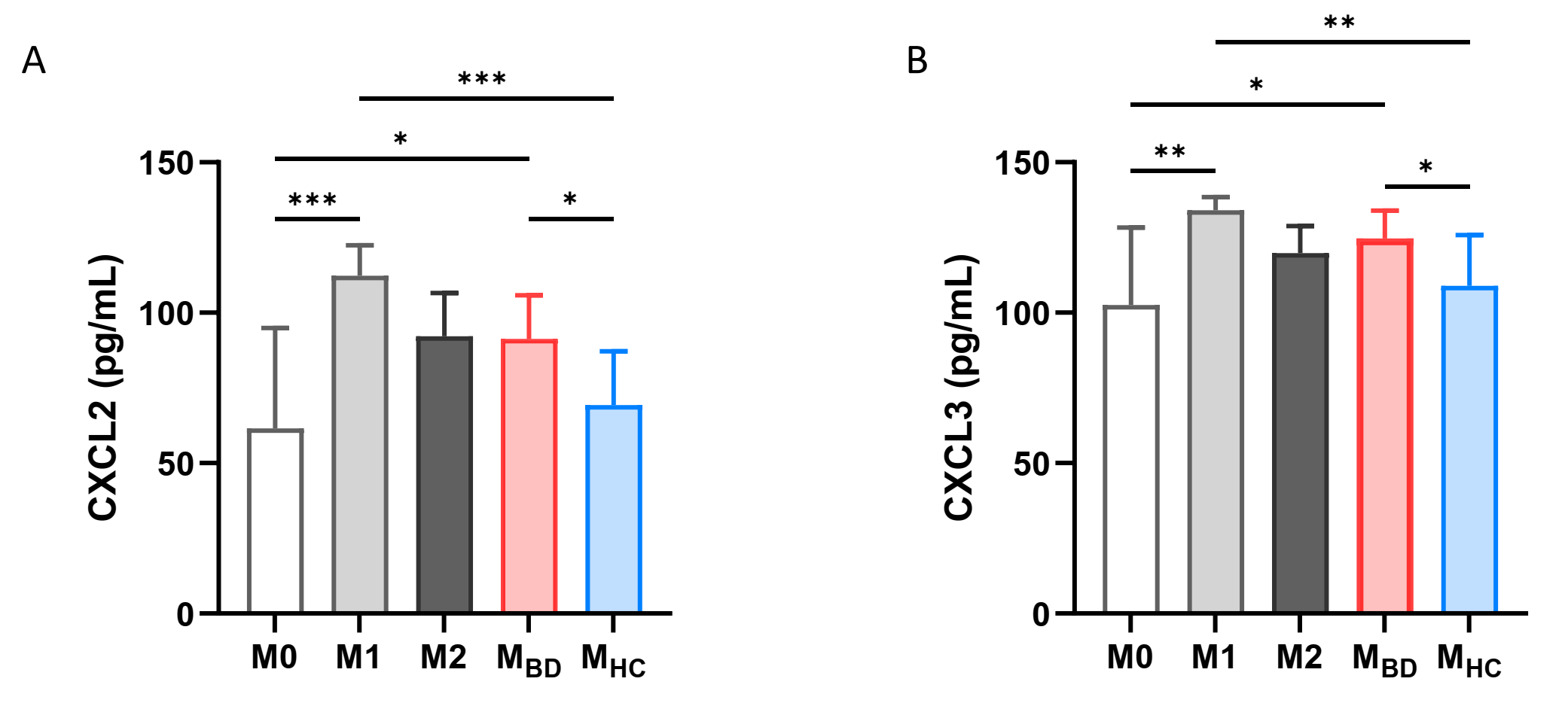


**Supplemental Figure S7.** **BD serum-treated macrophages produced more CXCL2 and CXCL3.**

Macrophages were stimulated with M0, M1 and M2 conditions, as well as BD serum and HC serum for 48 hours and supernatant were harvested for ELISA.

(A-B) CXCL2 and CXCL3 production by macrophages stimulated with M0 (n=6), M1 (n=6) and M2 (n=6) conditions, as well as BD (n=12) serum and HC (n=12) serum. Data were shown as mean±SD. *, p<0.05; **, p<0.01; ***, p<0.001; by One-way ANOVA. M_BD_, BD serum- treated macrophages; M_HC_, HC serum- treated macrophages.

**Supplemental Figure S8. NF-κB inhibition attenuated CD86 expression on macrophages stimulated by BD serum**

Macrophages were pretreated with DHE and then were stimulated with BD serum for 48 hours.

Summary graph of CD86 MFI level on DHE-treated and untreated macrophages stimulated with BD (n=6) serum and HC (n=6) serum were shown. Data were shown as mean±SD. **, p<0.01; ***, p<0.001, ****, p<0.001 by Two-way ANOVA.


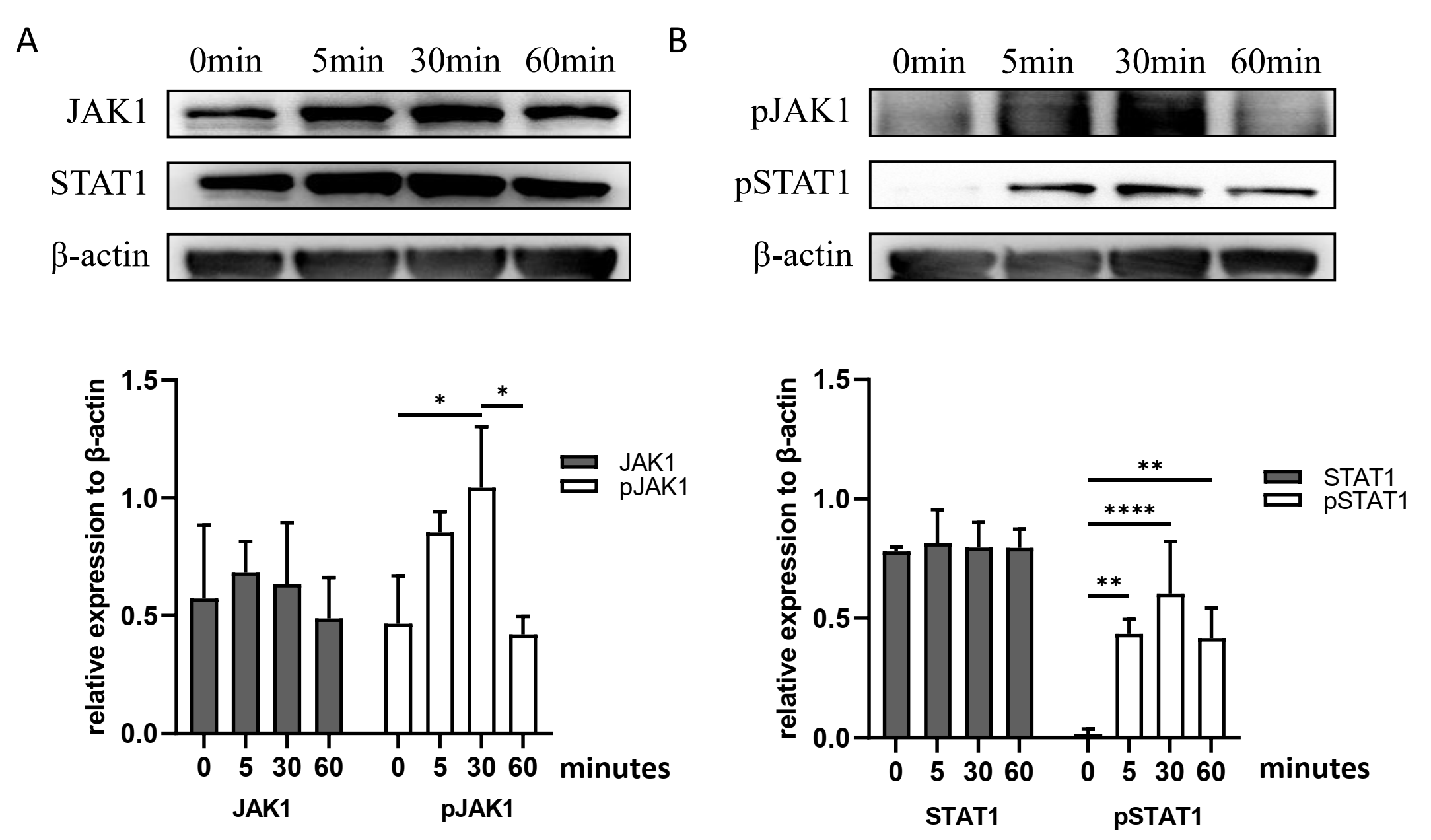


**Supplemental Figure S9 Activated JAK/STAT pathway in BD serum-treated macrophages.**

(A) Representative western blot images (upper) and summary (lower) of (A) JAK1, STAT1; (B) phospho-JAK1 and phospho-STAT1 of macrophages treated with BD (n=3) serum or HC (n=3) serum.

Data were shown as mean±SD. *, p<0.05; **, p<0.01, ****, p<0.001 by Two-way ANOVA. M_BD_, BD serum- treated macrophages; M_HC_, HC serum- treated macrophages.
